# “A Million Miles From How They Used to be”: Understanding Conversations From the Perspective of People with Dementia, Family, and Professionals

**DOI:** 10.1177/14713012251389447

**Published:** 2025-11-15

**Authors:** Alex Stirling, Catrin Hedd Jones, Suzanne Beeke

**Affiliations:** 1 Department of Language and Cognition, 4919 University College London (UCL), London, England; 2 Association for Dementia Studies, 8709 University of Worcester, Worcester, England

**Keywords:** dementia, conversation, community-dwelling, training, wellbeing, person-centred care

## Abstract

Dementia causes conversational difficulties for the person and those close to them due to the ubiquity of language and cognitive problems. Little is known about the experience of conversations from the perspective of people with dementia and their conversation partners (CPs). This study aims to understand experiences of conversations through consultation with people with dementia, those close to them, and dementia professionals, and how people are supported when they experience difficulties communicating. Nine people with a range of dementia diagnoses and six CPs (spouses), all community-dwelling, attended an in-person focus group in one of two locations. Sixteen dementia professionals attended online focus groups. Focus groups were recorded and analysed using reflexive thematic analysis. Four themes were identified: the impact of conversation changes for the person with dementia and CP, conversation and dementia identity, support for conversation, and conversation and person-centred care. Language and memory changes impacted conversations and relationships. Societal stigma also affected conversations. Support for conversation was perceived to be scarce. The need for education and training to equip families to maintain conversation was emphasised. Supporting conversation was seen as fundamental to maintaining relationships and as a mechanism for professionals to deliver person-centred care.

## Introduction

The number of people living with dementia is estimated at more than 944,000 in the UK alone (Dementia Statistics Hub, 2024). Language difficulties are common across the dementia spectrum (Suarez-Gonzalez et al., 2021) and in Alzheimer’s disease, word finding difficulties can be an early symptom (Forbes-McKay & Venneri, 2005). All areas of language function may be affected, including understanding of spoken language, word finding and sentence construction (Themistocleous, 2024), which impact a person with dementia’s ability to engage in conversations. In addition, the impact of cognitive changes on conversation can be profound. Difficulties with memory and attention can cause the person to ‘lose track,’ to repeat themselves and to struggle to follow conversations (Dijkstra et al., 2004).

Despite the ubiquity of language and cognitive difficulties in dementia, there is little research exploring perspectives of individuals with dementia in relation to their everyday conversations. Dementia research often excludes people with difficulty communicating (Murphy, Gray, van Achterberg, Wyke, & Cox, 2010). A systematic review by Alsawy et al. (2017) of experiences of communication, identified only one study (Day et al., 2011) that included the person with dementia’s perspective, rather than of family carer or healthcare professional. In response, Alsawy et al. (2019) interviewed individuals with dementia about what made communication meaningful. The authors found people appreciated efforts by family members to engage with them, as this increased feelings of connection. Conversely, being pressurised to produce ‘correct’ responses caused withdrawal from interactions. Volkmer, Bruns, et al. (2023) explored the experience of everyday conversations for people with dementia and their close CPs. They found that people attempted to make sense of the experience of dementia through interaction and reflected on how dementia caused changes in everyday conversation.

For individuals with dementia, participation in conversations may be affected by societal perceptions and assumptions (Nguyen & Lee, 2020). A person with dementia may be communicated with differently or even avoided (Milne, 2010). Societal stigma may be internalised, with the person becoming self-critical, causing reduced confidence and social withdrawal (Burgener et al., 2015). Family members may also experience stigma, and another consequence may be delay in seeking help (Nguyen & Lee, 2020).

Conversation is vital for psychosocial wellbeing, and people with dementia are likely to have fewer opportunities to experience this. Moyle et al. (2011) asked people with dementia about loneliness, and findings emphasise the importance of maintaining close, longstanding relationships. A move to a care setting is likely to severely weaken these bonds, highlighting the need to empower close family and friends to maintain conversations and connection.

Conversation difficulties also impact family members and are a significant contributor to caregiver burden (Savundranayagam et al., 2008). Communication changes are linked to higher rates of carer stress, depression, and isolation (Braun et al., 2010). Families often struggle to navigate their emotional responses to communication changes with their loved one with dementia, while simultaneously having to take a central role in supporting them (Braithwaite Stuart et al., 2022). The changes to a person as a result of dementia, and to conversations, have an impact on intimacy and relationships, which can cause deep loss (Purves & Phinney, 2012).

The UK National Institute for Clinical Excellence guidelines regarding dementia assessment, management, and care (NICE, 2018), state carers should be offered training involving psychoeducation and skills development. The guidelines make no recommendation about who should deliver training. Since speech and language therapy dementia provision is often limited (RCSLT, 2024), there is a need to recognise the role of other dementia professionals. Indeed, the Allied Health Professionals Dementia Framework for Wales (2022), advocates for multidisciplinary delivery of dementia education and training. Speech and language therapists (SLTs), feel their expertise in conversation support for dementia is not recognised (Bourgeois et al., 2016), while acknowledging that the profession has not promoted this role sufficiently. This research has been led by Speech and Language Therapists and a Psychologist.

Stanyon et al. (2016) asked a mixed group of professionals what facilitates communication in a care setting. They emphasised that building a relationship with a person with dementia was fundamental. They felt that training for professionals should go beyond communication strategies and address psychological and emotional aspects of dementia, for example attempting to take the perspective of the person with dementia and considering how one’s personal attributes influence relationship building. Stanyon et al.’s (2016) findings are echoed by Alsawy et al. (2017), where professionals again emphasised a strong relationship with the person with dementia, and maintaining ‘personhood’ (Kitwood, 1997) through valuing and respecting the person.

Education and training for CPs is a growing focus of research. Folder et al.’s (2024) systematic review of communication partner training for families of people with dementia aimed to identify intervention characteristics that might enable real life implementation of strategies by people with dementia and families. Across 30 papers there was improvement in families’ knowledge about dementia and in communication effectiveness. Participants described having more enjoyable conversations, and also reported positive impact on their relationships, with increased connection and engagement. However, they found mixed psychosocial outcomes, with no reduction in depression or anxiety for family carers or people with dementia The authors critiqued the heterogeneity of intervention approaches, and lack of clarity regarding intervention components and outcome measures, making clinical application problematic. Moreover, only seven of the studies mentioned any ‘consumer involvement,’ and the nature of this was not clarified. Ngyuen et al.’s (2018) systematic review of CP training in dementia for professional carers and family members reported improvement in communication skills and knowledge about dementia in all of the 17 studies. However, the findings for family CPs are difficult to isolate in this review. A review by Morris et al. (2017) produced similar findings, and also concluded that psychological impact for both person and carer requires targeted intervention.

Conversation between a person with dementia and a family carer is rarely a specific focus of intervention. There is a paucity of research targeting personalised intervention, which incorporates the communication skills and interests of both individuals in a conversational dyad (Kindell et al., 2017). However, two recent studies have focused on delivering individualised interventions. Training a dyad in the context of primary progressive aphasia (PPA), a rare language-led dementia, was carried out by Volkmer, Walton, et al. (2023) in their randomised control pilot study of Better Conversations with Primary Progressive Aphasia. This training focuses on reduction of barriers and on facilitatory strategies to support flow and enjoyment of conversations. Demonstrable changes in conversation were achieved, and therapy was rated positively. Morris et al. (2024) evaluated an individualised experiential course, Empowered Conversations (Morris et al., 2018). This training encourages care partners to understand the perspective of their family member with dementia, using empathic curiosity and reflect on their own communication through peer group discussion. The authors reported that participants felt better equipped in terms of strategies, and more able to connect with their partner living with dementia, with a positive impact on well-being and relationships.

The current study has a focus on exploring issues of dementia support for communication in urban and rural communities in England and Wales. Research contrasting the experience of dementia in these contexts has revealed particular issues in rural areas, including reduced access to specialist dementia support (Arsenault-LaPierre et al., 2023). Service provision in the native language of a person with dementia is also a significant issue. Clare et al. (2016) found Welsh speakers accessed dementia services later and with more advanced dementia. Stigma and shame regarding dementia in Welsh speaking communities were additional factors, with people less likely to ask for formal support, and presenting more often in crisis (Jones, 2018).

## Aim

This study aimed to understand the experiences of conversations through consultation with people with dementia, those close to them, and dementia professionals, across two contrasting areas geographically, demographically, and linguistically (London and North Wales). It also aimed to explore the support people have access to when they experience difficulties communicating.

## Methods

The study used a qualitative focus group methodology, which enables participants to discuss their experiences and respond to the views of others (Krueger & Casey, 2014). A group dynamic enables deeper and richer insights than individual interviews (Coenen et al., 2012). Care was taken to ensure participation was maximised for all, and information presented in an accessible way (Jones et al., 2021).

### Recruitment

Purposive recruitment targeted people with dementia, family members and friends, and people working or volunteering in the dementia field, all of whom expressed a specific interest in communication and conversation. The inclusion criteria for people with dementia were; any age, any dementia diagnosis, with mild or moderate dementia. This ensured they could understand the topic and participate in a group discussion about communication and conversation. Any family member or friend of a person with any diagnosis or severity of dementia was eligible. Thus they did not need to be relatives or friends of the people with dementia who took part. Any professional working or volunteering in the dementia field could participate.

Recruitment in London and North Wales was through two community organisations for people with dementia. Staff were gatekeepers and identified people and family members who could agree to participate. In North Wales, participants were offered the choice to attend a focus group conducted in English or Welsh. All project information followed guidelines for dementia accessibility (DEEP, 2024) and was available bilingually in Wales. Professionals working or volunteering in dementia services were recruited through a voluntary organisation in London and a dementia professionals’ group in North Wales. Project information was also circulated by the Dementia Lead for Allied Health Professionals in Wales. Paper copies of consent forms were completed in advance by participants with dementia (supported by group coordinators if needed), or at the beginning of the focus groups. Professionals completed online consent forms in advance.

### Participant Information

Demographic information was collected as follows:

• Person with dementia: age, gender, dementia type if known, how long living with dementia.
• Relative/friend of a person with dementia: age, gender, relationship to a person with dementia, dementia type if known, how long living with dementia.

 Participants in North Wales were also asked about their preferred language for verbal and written correspondence.

• Professionals: age; gender; job title; job sector; how long working/volunteering in dementia.

### Focus Group Procedures

Focus groups for people with dementia and their CPs (one in London, and one in North Wales), were conducted in-person, lasting 90 minutes. Two focus groups for professionals were held online. Each group was facilitated by the lead author (a speech and language therapist) and an agenda provided using guidelines for accessibility (DEEP, 2024). A topic guide was developed, covering how conversations with dementia are experienced (what helps, what is difficult), what support individuals receive or provide, and what support would be available in an ideal world. Focus groups were video recorded; in person by video camera and online using Microsoft Teams. Transcriptions were made, checked and anonymised. All identifiers were removed, and specific job roles made generic.

### Data Analysis

Data were analysed using reflexive thematic analysis (Braun & Clarke, 2019). This qualitative approach is suited to analysis of focus group data. It acknowledges the particular standpoint of the researcher and what they bring to the analysis process, in terms of context, personal experience and professional background. The lead author/AS is a speech and language therapist, with a clinical specialism in conversation-based therapy approaches. She has experience of dementia in her family and awareness gained from the lived experience of people with dementia and families that support for conversation is often limited or non-existent. The second author is a psychologist raised within a Welsh speaking community and aware of the lack of support in native languages. As lecturer and researcher in dementia she has worked alongside experts by experience to educate health care students and researchers on how best to support people to live their best possible lives. The final author is. an academic speech and language therapist with a specialism in conversation, and has experience of dementia in her family. 

As speech and language therapists and a psychologist, the authors’ concept of communication is underpinned by linguistic and psychological knowledge of communication as both transaction and interaction. We view it as inherently multi-modal, involving verbal and non-verbal behaviours. We conceptualise communication as social interaction and thus linked to wellbeing.

The goal of reflexive thematic analysis is to tell a story creatively and flexibly, while being transparent about the subjectivity of the analyst but also the rigour of the process. There are six stages of the analysis process; (1) familiarising yourself with the dataset, (2) coding, (where brief labels that capture important features of the data are generated) (3) generating initial themes, (4) developing and reviewing themes, (5) refining, defining and naming themes, and (6) writing up (Braun & Clarke, 2019). The lead author was responsible for stages 1, 2 and 3. All authors were involved in developing and reviewing themes, refining, defining and naming themes and in writing up, ensuring the credibility and trustworthiness of the thematic analysis process. The data were integrated and analysed as one large data set, in order to gain understanding of the collective experiences and views.

## Results

### Participants

In London, four people with dementia and two family members attended the initial focus group. The two family members were spouses of people with severe dementia not involved in the study. A further two people with dementia who were unable to attend the first meeting attended a separate group one week later. In North Wales, six participants attended the focus group in English: three people with dementia and three family members (spouses) of other individuals with dementia. Only one Welsh speaking person (a spouse) attended the Welsh medium group and therefore this became an interview, conducted by the second author. All participants were community-dwelling.

See Table 1 for demographic information.

**Table 1.** Demographic Information: Participants With Dementia (PWD) and CPs

Participant	Gender	Age	Dementia type	Time living with dementia (person or their spouse)
London				
PWD 1	F	86	Unsure/undisclosed	Unsure/undisclosed
PWD 2	F	82	Alzheimer’s disease	I year
PWD 3	F	75	Unsure/undisclosed	Unsure/undisclosed
PWD 4	F	81	Unsure/undisclosed	Unsure/undisclosed
PWD 5	F	62	PPA	1 year
PWD 6	M	75	Dementia with Parkinson’s disease	5 years
CP 1	F	71	Spouse with Alzheimer’s disease	7 years
CP 2	M	79	Spouse with Alzheimer’s disease	8 years
North Wales				
PWD 7	F	86	Alzheimer’s disease	1 year
PWD 8	F	72	Unsure/undisclosed	Unsure/undisclosed
PWD 9	M	64	Fronto-temporal dementia	4 years
CP 3	F	76	Spouse with vascular dementia	1 year
CP 4	F	72	Spouse with Alzheimer’s disease	I year
CP 5	F	80	Spouse with Alzheimer’s disease	3 years
CP 6*	F	68	Spouse with vascular dementia	10 years

NB: Four of the people with dementia were unable to state what kind of dementia they had or how long they had been living with dementia. While the group coordinators did have access to this information, the ethical approval granted did not cover sharing this with the researcher. Therefore, unsure/undisclosed has been entered for these participants.

Due to a high degree of interest, two online focus groups for professionals were conducted, with a total of 16 attendees, 11 working in Wales, 5 in England. See Table 2.

**Table 2.** Demographic Information: Professionals

Job role	Code	Gender	Age (range)	Work sector	Years experience
Dementia activity support Worker	P1	F	45–54	Health care	<2 years
Dementia activities coordinator	P2	M	35–44	Voluntary sector	>10 years
Supporting health & wellbeing manager	P3	F	45–54	Social care	2-5 years
Wellbeing manager	P4	F	45–54	Voluntary sector	2-5 years
Dementia coordinator for a local council	P5	F	55–64	Social care	5-10 years
Dementia active community officer	P6	F	25–34	Social care	2-5 years
Personal assistant/Carer	P7	F	45–54	Social care	2-5 years
Speech & Language therapist	P8	F	45–54	Health care	>10 years
Speech & Language therapist	P9	F	45–54	Health care	>10 years
Speech & Language therapist	P10	F	45–54	Health care	>10 years
Clinical psychologist (& volunteer)	P11	F	55–64	HealthCare	2-5 years
Assistant psychologist	P12	F	25–34	Health care	<2 years
Dementia practice educator	P13	F	45–54	Health care	>10 years
Occupational therapist	P14	F	45–54	Health care	2-5 years
Occupational therapist	P15	F	35–44	Health care	<2 years
Nurse specialist with young onset dementia	P16	F	45–54	Health care	>10 years

## Key Findings

There are four key themes: the impact of conversation changes for the person with dementia and the CP; conversation and dementia identity; support for conversation; conversation and person-centred care. Themes 1, 2 and 3 relate to all three groups interviewed, whereas Theme 4 is specific to dementia professionals.

### Impact of Conversation Changes for the Person with Dementia and the CP

Language and cognitive changes affect conversation, with resultant emotional impacts and changes to relationships and roles. The changes that people with dementia and CPs report implicate both language and cognition. People with dementia and partners are aware of difficulties in both understanding language and finding words: ‘*You can’t sometimes say the word…and sometimes I say, ‘I don’t understand’ (PWD5)*. ‘*I say something, and it will come out back to front*’ *(PWD8)*. They notice that difficulties worsen over time: *‘his language has become very poor’ (CP1)*.

A problem with names is an early symptom: *‘The first signs of dementia were 10 years ago when she started having difficulty recalling names. And difficulty recalling names quickly developed into difficulty finding the right word’ (CP2)*. A professional carer describes increasing language problems for the person she supports: ‘*Words have become less and less, sentences even more so’ (P7)*.

Memory problems are pervasive. People with dementia may lose track or be unable to remember conversations: *‘I will…start saying something and then it may have gone*. *I’ll start answering a question or completing a point …and the world has moved on’ (PWD6)*. *‘I am able to have a conversation, but I won’t remember it’ (PWD3)*. CPs describe spouses repeating themselves: ‘*Conversation is one thing that isn’t a problem with my husband*. *He will sometimes give you the same conversation more than once*’ *(CP4)*. Professionals are also aware of cognitive problems, in one case linking memory to reduced insight: *‘They say, “I don’t have any communication difficulties, I’m fine*.*” Because obviously they might forget that they might not understand what we’re talking about’ (P2)*.

The emotional impact of these changes is particularly evident for the participant with PPA, who fears the progression of her language problems, and grieves identity change: *‘You’re all going to be ok, you’re all gonna be able to talk’* and *‘I’m a very chatterbox’ (PWD5)*. Memory problems cause anxiety. A person with dementia wakes at night to check text reminders that follow conversations with her children *(PWD3*).

Frustration is prevalent and noted by all three groups: *‘It’s the frustration that I can’t tell you and it’s getting worse now when I’m tired’ (PWD5)*. *‘He gets frustrated because he knows what he wants to say but he hasn’t got the words’ (CP1)*. *‘Some people also hit themselves and say “‘no, my head is not bringing the words’” (P7)*. Partners also feel great frustration themselves: *‘It’s been impossible trying to work out what she’s trying to say’ (CP2)*, and ‘*enough, I’ve answered it 20 times!’ (CP1)*. A professional observes the impact on both: *‘I think communication, this frustration with communication between the carer and the person with dementia, that’s a difficulty’ (P14)*.

Social withdrawal is reported: *‘You become less sociable*. *You become more conscious of er, overcoming words and such like’ (PWD6)*. *‘It’s a total loss of confidence; it takes him a while to digest’ (CP3)*. ‘*If you have a conversation together, it’s not that we intend to dominate the conversation*. *But you just do because the man got very quiet’ (CP3)*. Professionals notice that ‘*they call themselves stupid*. *Sometimes it impacts on their confidence to go out into social groups, and they just want to hide away’ (P16)*. People may now view themselves negatively: *‘It’s a waste of time talking to me, I can’t remember anything’ (PWD3)* and *‘you need people with patience around you, yeah, to listen through the garbage’ (PWD6)*. *‘I repeat myself…which is a bit boring for other people’ (PWD4)*. *‘I’m feeling so thick, I can’t’ (PWD5)*.

Spouses report having to organise conversational opportunities for their partners: *‘His friend takes him to the pub on a Friday night with his brother, and he comes [to the group] on a Tuesday*. *And whatever else I fill in as best I can’ (CP4)*. Encouraging communication is an extra responsibility: ‘*It’s such a frustrating existence for all of us in this, it’s just adding to the burden’ (CP5)*. One person with dementia is aware of the additional demands on his partner: ‘*When we want to communicate with the outside world, she has to deal with all that’ (PWD6)*.

Changes to conversation lead to changes in relationships, with loss of intimacy and resultant isolation. A professional observes that a person with dementia and their CP ‘*can’t talk openly or have those loving conversations, and that just has a massive impact on the relationships’ (P5)*. She sums up the impact: ‘C*onversation is a million miles from how they used to be…you can feel the pain they’re both going through*.*’* She adds: ‘*they’ve got these soul mates and all of a sudden they’re having … conversations that are just, they’re just not happening*.*’* A speech and language therapist (SLT) reports being told by a spouse: *‘you’re asking me to change the way I’ve communicated with my husband for 50 years’ (P8)*.

### Conversation and Dementia Identity

Experiences of conversations and dementia are affected by issues beyond language and memory, encompassing stigma and the imposition of a dementia identity. Societal stigma surrounds dementia, and this can impact interaction. School children visiting a group confess they are ‘scared’ to engage in conversation. But as the group coordinator comments: *‘I see them like laughing and smiling like the same chat you will have with your grandfather, your grandmother’* and they say after: “*I had a really good time, not that it was OK, but I loved it!”’ (P2)*.

Stigma may contribute to denial and rejection of dementia by the person and those close to them. A person with dementia says: ‘*I’m not sure I’ve got dementia to be honest’ (PWD1),* though possible lack of insight must also be acknowledged here. One partner reports that her husband with dementia would not attend the focus group for this study: *‘It would have made him agitated because I have never used the word dementia with him*. *He got so upset about it at the beginning and then I did not mention it, maybe I should have, and we would be able to discuss it more openly now… He did not want to talk at all about the dementia, in real denial’ (CP6, translated)*.

One professional feels that denial is ‘*a huge issue*’ for families, directly affecting communication: *‘Very often it’s yes, they hear, they get the education, their head can hear us, but their emotional response in the moment and when the difficulties occur is really sort of, ‘no he’s just being difficult’ (P9)*. A professional views acceptance as a prerequisite for improved communication and conversation: *‘It is about families learning to accept the situation and going on to learning how to communicate better’ (P8)*. However, professionals may avoid the word dementia themselves: *‘In practice I sometimes try and avoid using the words dementia and kind of mention the particular issue* [the person is having with communication]’ *(P10)*. This participant feels it is ‘*emotionally charging to use the word dementia*.’ Another professional says: *‘I say to someone, um, “what about memory loss?” I will try to avoid dementia and Alzheimer’s words’ (P2)*.

Dementia means people may be viewed through a specific lens and allocated a new identity. They may be seen as fundamentally changed. A professional describes a state of liminality; a person with dementia caught between two identities: ‘*She seems to be in a chrysalis now, she’s not the caterpillar she used to be, but she hasn’t come out as the butterfly…while she’s kind of in this cocoon they’re really struggling to communicate with each other’ (P5)*.

A partner is bewildered by a fundamental change to identity and language use: ‘*We are so Welsh and everyone in the family speaks Welsh, and he has started to talk to me in English’ (CP6, translated)*. Change may be resisted by those close to the person: *‘They [friends] just want M [person with dementia] to be M’* (*P7)*. This view of changed identity impacts conversation and social engagement. Participants notice that people with dementia can be infantilised. A professional describes: *‘one of my pet hates, when people refer to the ‘little old lady*.*” Now, it’s incredibly patronising’* (*P3*). Relatives may express incredulity at memory difficulties. A person with dementia remembers her children laughing at her mother (who also had dementia): ‘*you’ve already said that!*’ *(PWD2)*. And a professional notices a tone of disbelief from family members: *‘“Are you serious, mum, you don’t remember*?”’*(P2)*. The person with dementia is no longer viewed as a reliable narrator. A spouse feels she has to monitor her husband’s phone calls: *‘Sometimes I’ll hear a conversation, and I’ll check it out*. *And it’s not quite what he says’ (CP4)*. A professional describes a person with dementia saying: *‘memory problems, me, no!’* and goes on to describe *‘the relatives behind the person doing signs saying, ‘yes, yes you have’’’(P2)*.

### Support for Conversation

People with dementia and families have received little or no professional conversation support. All groups emphasised the need for input early and throughout. Other factors that support include positive environments and families altering their communication.

Most people have not encountered professional support for communication and conversation difficulties: *‘Nobody even mentioned going anywhere for speech at all’ (CP4)*. *‘I never thought to ask about communication’ (CP 6)*. Even when help is sought from a doctor or memory clinic, people have sometimes been dismissed: *‘His speech was quite bad at that point really, but they put it down to anxiety’ (CP4)* and ‘*there’s nothing we can do for you, come back when it gets much worse’ (CP2)*. Only one participant, who has PPA, has received speech and language therapy for communication. She found this helpful, but funding was cut. There is ‘*nowhere to get speech and language therapy’* and she has been ‘*begging for speech and language therapy’ (PWD5)*. A partner wonders at the reason for lack of this support: ‘*Is it because they think, well, they’re not going to remember if they could speak, you know if they had therapy, they wouldn’t remember it?*’ *(CP4)*.

One of the SLTs feels that ‘*speech therapists don’t see the role if someone’s got cognitive difficulties, if it’s not aphasia* [a non-progressive communication difficulty caused by stroke] *or a language problem’ (P8)*. One professional describes that speech and language therapy is for *‘swallowing assessments, mostly where I am, and maybe not so much for communication’ (P16)*.

Professionals emphasise throughout that *‘families need support, training, ideas’ (P2)*, to give the person with dementia a better quality of life and to provide ‘*tools to stop people having no conversation’ (P3)*. The link between equipping families to support conversation and a person with dementia’s wellbeing is emphasised: *‘It’s extremely important for people with dementia to keep a good mental health, and as part of this, family need to know exactly how to maintain a good conversation with people with dementia’ (P11)*.They feel families lack awareness and education about dementia: *‘A lot of the communication difficulties is partly because lots of family members don’t understand the condition’ (P4)*. Families report of the person with dementia: *‘oh they’re being repetitive’ or ‘they’re saying things that aren’t true’* (*P15)* or that they are *‘just being difficult*. *They say that their person is playing games, they’re doing it on purpose or they’re lying’ (P2)*.

Early support for conversation is emphasised, to enable the person and family to have most chance of using strategies successfully: ‘*I’m going to get some help and start it early*’ *(PWD5)***.** One spouse comments sadly: *‘we are long past those stages; it’s too late for us now’ (CP2)*. Early support means you ‘*build that relationship and you’re not left really until the point of crisis to be able to understand how to communicate better*.*’ (P3)*. *‘The difficulties don’t happen the day before somebody presents in the clinic, it’s been happening for years potentially’* (*P16)*. Support throughout is emphasised: *‘It is really important that people are able to access that help at any point’ (P2)*.

People with dementia describe how environments can support their conversations and relationships: ‘*I think being amongst people helps, because being isolated doesn’t help*. *It’s a nice atmosphere [the day centre]*. *It’s good, we talk to each other’ (PWD2)*. *‘We are in the same boat*. *There’s understanding, and nobody judges you*.*’ (PWD4)*. People with dementia, spouses and professionals all report the importance of allowing time: *‘The biggest thing is time…so, my friends I say, ‘you have to wait you know*. *(PWD5)*. *‘Time it’s just giving time’ (P7)*. Partners change their communication to support conversations, for example, asking questions and keeping their speech simple: *‘I said, can you take your socks off? And he’ll take his top off*. *And I’ll say, no, how about your socks? They’re on your feet*. *‘Where are my feet?’ …*..*and if I say, take your slippers off, he’ll say ‘what, all of them?’ (CP1)*. *‘I have to ask him questions, so* ..*is it these? Or your teeth? Cos his teeth go missing too*. *I just have to quiz which bit of the anatomy we are talking about’ (CP3)*. *‘I’ve just learned not to bombard, yeah’ (CP4)*.

### Conversation and Person-Centred Care

Professionals view conversation as the foundation of person-centred intervention: ‘*I’m on a mission to kind of promote chatting, cos that’s how we build relationships*.*’ (P8)*. This SLT perceives that people with dementia are increasingly denied social contact because communication becomes transactional: *‘Communication moves from kind of a relationship and chatting and that sort of social aspect to a more needs led communication as the dementia advances*.*’* Another professional feels that good communication is ‘*the basis for everything… we need to make sure that the way that we’re communicating with people helps them to engage in those things that are meaningful’ (P10)*.

Conversation is seen by professionals as the mechanism by which a therapeutic relationship is established: *‘When you connect like that, that’s priceless, because they feel like they’re being listened to*. *They feel they’ve got something important, something valid to say*.*’ (P16)*. The need to really listen to the person with dementia is also emphasised, in order to try and connect: ‘*Listening is very important because sometimes people may be using words, but they’re actually communicating something different*’ (*P10*).

Conversation enables professionals to adopt an individualised, person-centred approach. *‘I get to know the people really really well, and every single person’s different as well’ (P16)*. Another professional concurs, emphasising that you need to *‘get a good understanding of them as a person*. *So we can make that person-centred*. *So when we go and engage with them, they’re more open*. *We can develop that relationship with them’ (P15)*. This person-centred approach is enacted through flexibility, and adapting communication according to particular needs and preferences: ‘*Sometimes it’s easier for her to communicate in English and I go with that if she wants to speak English…so, you just go with the person?*’ *(P7)*. A SLT echoes this personalised approach: *‘You can’t use the conveyer belt approach at all’ (P8)*. She sees her role as reinforcing individual strategies that families have already adopted. ‘*What’s working now, rather than going in straight way and giving advice*’ and another SLT agrees: ‘*Reassuring and saying, well, that’s working for you and that’s great, so carry on with that’ (P10)*.

## Discussion

This study highlights the impact that dementia has on everyday connections. Conversations and relationships are impacted by language and memory changes in dementia, as well as societal stigma and the imposition of a dementia identity. Support for conversation is perceived to be scarce. The need for education and training to equip families to maintain conversation is emphasised. Supporting conversation is seen as fundamental to maintaining relationships as well as a mechanism for professionals to deliver person-centred care.

People with dementia report frustration, loss of confidence and social withdrawal due to awareness of their difficulties. They may express anxiety about the future, and awareness of the impact on those close to them. CPs (here spouses) express their struggles with communication changes and the impact on conversations, relationships, and intimacy. Professionals emphasise the frustration they observe in communication between a person with dementia and their family, and how relationships are negatively impacted. The need for education and training around communication and conversation is emphasised.

Stigma regarding dementia is clearly represented here. People with dementia may be viewed differently, negatively affecting interactions. They sometimes express negativity and self-criticism, which may be internalisation of societal stigma, as described by Milne (2010). Stigma appears particularly present here among first language Welsh speakers, resulting in apparent reluctance to attend the focus group and even to acknowledge a dementia diagnosis in some cases. The underlying reasons for this were not probed.

Linked to this, professionals in this study avoid the word dementia, due partly to perceived emotional impact on person and family, echoing the findings of Kissel and Carpenter (2007). Their interviews with ten physicians experienced with dementia showed great variability in the disclosure of a dementia diagnosis, including sometimes using ‘memory problems’ or ‘dementia’ rather than ‘Alzheimer’s disease’ due to concern that the latter term may evoke hopelessness and fear of the future for person and family. Professionals were found to be more likely to use euphemistic terms (e.g. memory problems, forgetfulness) rather than medical terms, again, due to perceived emotional distress or stigma (Caruana-Pulpan & Scerri, 2014). While stigma may be a factor in professionals avoiding the word dementia (Phillips et al., 2012), perhaps avoidance actually perpetuates stigma, since research has shown that limited knowledge about dementia makes stigmatising attitudes more likely (Herrmann et al., 2018). It is interesting to reflect that, for aphasia, a non-progressive communication difficulty commonly caused by stroke, people reflect negatively on the fact that the term aphasia was not clearly explained to them, or in some cases not even mentioned (Worrall et al., 2011). Information is a key priority for people with aphasia, as one person commented ‘*Once you’ve got a name for something, it’s like you’ve got half the problem sorted…You mightn’t be able to cure it and everything else but you can understand it more*’ (Worrall et al., 2011, p.314). Education regarding dementia is surely equally important and could be addressed with direct but sensitive communication that focuses on the individual experience, and challenges generalisations and stereotypes (Harper et al., 2019).

CPs and professionals give a strong message about the lack of education and training support for conversations. The people with dementia do not mention education and training, which may relate to the internalisation of societal stigma, leading them to locate the ‘problem’ with themselves rather than with society (Milne, 2010). Even when people with dementia and families sought help, it was often not provided, and the professionals they consulted appeared not to know what support might be available. This echoes the findings of Volkmer et al. (2019) that professionals lack awareness of the role of speech and language therapy in supporting communication in dementia (here PPA). Professionals in the current study strongly advocate for education and training to equip those close to the person with tools to maintain conversations, and thus relationships and wellbeing. They perceive that family members lack understanding about dementia, causing them to misinterpret communication, with the person with dementia seen as simply being ‘difficult,’ for example. These professionals feel that family members’ acceptance of a dementia diagnosis necessarily precedes the possibility of changing their own communication. Psychological adjustment for carers is thus a vital part of enabling positive conversations and relationships, for example through empathy and self-reflection, as described by Morris et al. (2018).

Professionals in this study view conversation as a mechanism to enable the formation of a validating relationship with a person with dementia. Fazio et al. (2018) in their review of person-centred care in dementia, emphasised the fundamental importance of a meaningful authentic relationship between the person with dementia and professional, established through communication that explores the person’s individual history, personality, and values. Webb (2017, p. 1105) makes the powerful point that everyday conversation ‘*is particularly pertinent to the aims of ‘person-centredness’, because it has long been argued that personhood is created via the process of interaction with others*’. This contrasts with the ‘needs led’ communication that is often a default in more advanced dementia. It emphasises the vital importance of maintaining social interaction, as a means by which a person with dementia can reveal their unique self and through which a meaningful relationship can be established (Blackhall et al., 2011).

### Study Limitations and Future Research

The CPs in these focus groups were all spouses and it was therefore not possible to gain a wider perspective from different close CPs such as other family members or friends. However, this may reflect reality, as spouses increasingly play a central role in their partner with dementia’s care and social opportunities (Brodaty & Donkin, 2009). It would be useful to explore the views of a wider group of relatives and friends, to see how these may compare to those of spouses. Although the study was not able to directly include the perspectives of people with more advanced dementia, several spouses described the experience of conversations with partners at this stage of dementia. The exploration of urban versus rural services, and the experiences of people who speak a minority language (Welsh) was more limited than intended. Although only one Welsh speaking CP took part, a strong proportion of the professionals were first language Welsh speakers.

It feels important to further explore the experience of stigma and the impact this has on conversations and social engagement, as well as on accessing support for communication. It is clear that professionals here view support for families’ emotional adjustment to dementia to be a vital part of communication training. This is exemplified by the communication empowerment framework training (Morris et al., 2018). However, as yet this is small-scale dyadic training between the person and their family member. There is clearly both a knowledge and implementation gap, and a need for awareness and availability of training on a larger scale.

In conclusion, this research adds to a small but growing body of work that has elicited the views of people with dementia about communication and conversations. Conversation is viewed as central to relationships and wellbeing in dementia, as well as a mechanism for the delivery of person-centred care. Support for conversation in dementia is perceived as scarce, and participants emphasise a vital need to equip CPs (particularly family members), with both emotional support and practical tools in order to maintain conversations and connection with their loved one with dementia.

## Supplemental Material

Supplemental Material - “A Million Miles From How They Used to be”: Understanding Conversations From the Perspective of People with Dementia, Family, and Professionals Supplemental Material for “A Million Miles From How They Used to be”: Understanding Conversations From the Perspective of People with Dementia, Family, and Professionals by Alex Stirling, Catrin Hedd Jones, Suzanne Beeke in Dementia

## Data Availability

The datasets generated and/or analysed during the current study are available from the corresponding author on reasonable request.*
