# Supplementary material for: “A Million Miles From How They Used to be”: Understanding Conversations From the Perspective of People with Dementia, Family, and Professionals
Source: Dementia (London). 2025 Nov 15;25(8):1879–95. doi: 10.1177/14713012251389447 (PMC13683889; doi:10.1177/14713012251389447)
Supplement: Supplementary file 1 [file sj-pdf-1-dem-10.1177_14713012251389447.pdf]

Focus Group: people with dementia and relatives/friends

Agenda	Prompts/Areas to cover
1. Introductions & Welcome	
2. Restate purpose of focus group and plan of session	
3. Brief accessible introduction about what we might mean re terms we use.<br><br>4. How you feel about conversations	*e.g., when I talk about conversations, I mean having a chat with others. It can be wider than just talking. It could be body language, facial expression, gesture, showing things, writing, drawing.*<br><br>*what does conversation mean to you?*<br><br>*What are conversations like for you with dementia?*<br><br>*How have they changed since dementia?*<br><br>*What works well/helps you?*<br><br>*What is hard?*<br><br>*What would you like to be doing with your conversations?*
5. What help have you had: if any	*Have you had any help? (advice, training, therapy etc).*<br><br>*If you've had any help, who was it from? (e.g.speech and language therapist, voluntary organisation, others experiencing the same thing, friends, online)*

	Where did any support or training happen? (e.g. at home, at a group, online)<br><br>*When did you get this help?*
6. In an ideal world.	*What help with conversations do you think might be the most useful?*<br><br>*What might have helped you at the beginning?*<br><br>*What might help now?*<br><br>*How could this best be provided? (person with dementia on their own, conversation partner on their own, together? group?)*<br><br>*Where might this best happen?*<br><br>*When would you want help?*
7. Conclusion	*Recap of session*<br>*Any questions?*<br>*Thanks, and goodbyes*

Online Focus Group: Professionals

**Agenda**	**Prompts/Areas to cover**
5. Introductions Welcome	*Thank you for coming*<br><br>*Introductions round group*
6. Restate purpose of focus groups and plan of session	*My background + rationale*<br>*Plan of session (share)*

7. Brief introduction about terminology<br><br><br><br><br><br><br><br><br>8. Impact of dementia on conversation	*Conversation, communication, talking?*<br><br>*Finding words?*<br><br>*What do you use with people with dementia and their families/friends?*<br><br><br><br>*What impact do you feel dementia has on conversation?*<br><br>*What do people tell you helps?*<br><br>*What do people tell you is hard?*<br><br>*What do people tell you they'd like to be doing in terms of conversation?*
5. What help if any do you provide in your setting?	*Do you provide any help? (advice, training, therapy etc).*<br><br>*If so what does it consist of?*<br><br>*Who does it ?*<br><br>*How? (e.g. person with dementia, family/friend, both, group)*<br><br>*Where does it happen? (e.g. at home, at your work, online)*<br><br>*When is help offered?*<br><br>*Biggest challenge for you?*
6. In an ideal world.	*What help with conversations do you think might be the most useful?*<br><br>*How could this best be provided? (e.g. person with dementia on their own, conversation partner on their own, together? group?)*<br><br><br>*Where might this best happen?*

	When would this help best be offered ?
7. Conclusion	*Recap of session*<br>*Any questions?*<br>*Thanks, and goodbyes*
